# Genetic Structure and Inferences on Potential Source Areas for *Bactrocera dorsalis* (Hendel) Based on Mitochondrial and Microsatellite Markers

**DOI:** 10.1371/journal.pone.0037083

**Published:** 2012-05-16

**Authors:** Wei Shi, Carole Kerdelhué, Hui Ye

**Affiliations:** 1 Laboratory of Biological Invasion and Transboundary Ecosecurity, School of Agriculture Research, Yunnan University, Kunming, People's Republic of China; 2 INRA, UMR CBGP, Campus de Baillarguet, Montferrier-sur-Lez, France; Sun Yat-sen University, China

## Abstract

*Bactrocera dorsalis* (Diptera: Tephritidae) is mainly distributed in tropical and subtropical Asia and in the Pacific region. Despite its economic importance, very few studies have addressed the question of the wide genetic structure and potential source area of this species. This pilot study attempts to infer the native region of this pest and its colonization pathways in Asia. Combining mitochondrial and microsatellite markers, we evaluated the level of genetic diversity, genetic structure, and the gene flow among fly populations collected across Southeast Asia and China. A complex and significant genetic structure corresponding to the geographic pattern was found with both types of molecular markers. However, the genetic structure found was rather weak in both cases, and no pattern of isolation by distance was identified. Multiple long-distance dispersal events and miscellaneous host selection by this species may explain the results. These complex patterns may have been influenced by human-mediated transportation of the pest from one area to another and the complex topography of the study region. For both mitochondrial and microsatellite data, no signs of bottleneck or founder events could be identified. Nonetheless, maximal genetic diversity was observed in Myanmar, Vietnam and Guangdong (China) and asymmetric migration patterns were found. These results provide indirect evidence that the tropical regions of Southeast Asia and southern coast of China may be considered as the native range of the species and the population expansion is northward. Yunnan (China) is a contact zone that has been colonized from different sources. Regions along the southern coast of Vietnam and China probably served to colonize mainly the southern region of China. Southern coastal regions of China may also have colonized central parts of China and of central Yunnan.

## Introduction

Determining the source area and understanding the colonization routes of invasive pests are key issues when developing management strategies [1], [2]. Introduced pest species are a major threat to the environment causing economic harm due to the negative consequences of their proliferation and the costs of controlling their propagation. Molecular genetics methods are powerful tools in tracing invasion patterns of introduced pests and inferring their potential sources. However, it can be difficult even to identify the native range of the target species because of the lack of historical data.

*Bactrocera dorsalis* (Hendel), the oriental fruit fly, is one of the most notorious species in the family Tephritidae. This species belongs to the *B. dorsalis* species complex [3], and is a highly polyphagous pest that attacks more than 250 plant species, including a number of commercially grown fruits such as melon, banana, mango and guava [4]. Because of its wide host range and *K*-selected demographic strategy [5], [6], it has been suggested that *B. dorsalis* would be adapted for growth and establishment in near optimal environmental conditions, taking advantage of the most productive niches [7]. It apparently can disperse very quickly. For example, this fly was first described in Taiwan [3], [8] in the beginning of last century. Over the following 90 years, it has apparently expanded throughout tropical and subtropical Asia and around the Pacific Ocean [8]. *B. dorsalis* has been regarded as a typical invasive species causing high economic losses, and many research programs have been already carried out in the fields of ecology and biology [9], [10]. Yet, little is known about its natural range, and the actual migration pathways between its potential native range and the recently colonized areas. It is evident that a detailed knowledge of the biology, geographical variability and genetic features is a prerequisite to plan strategies for quarantine, control or eradication of any pest species [11]. Concerning genetic studies, most papers focused so far on molecular taxonomy and species delimitations within the species complex [12]–[14] or on intraspecific genetic structure at regional scales [15]–[20]. These preliminary data suggested that the fly had high levels of genetic diversity even at a local scale, and showed very little genetic structure. Based on these results, Shi et al. [17], [18] even hypothesized that Yunnan could be within the source area of this fly, or that the fly colonized Yunnan a long time ago.

The question of identifying the source of origin and understanding the colonization pathway for this species has received less attention. Recently, two studies focused on this issue. One paper was based on microsatellites only [7], with samples from South Asian and Southeast Asian countries (Bangladesh, Myanmar, Laos, Thailand and Cambodia), and from one single population in mainland China (Guangdong), where only 9 individuals were sampled. This work showed that the genetic structure of *B. dorsalis* in these regions is complex, and its interpretation was not straightforward. Some populations at the western limit of the range (Bangladesh, Myanmar) or in which the fly was recently introduced (Taiwan, Hawaii) were somewhat differentiated, while high gene flow was suggested among Southeast Asian countries, namely Laos, Thailand and Cambodia. Aketarawong et al. [7] suggested that Far East Asia could be the source area for this fruit fly, which would have then migrated westward. These conclusions were limited by a lack of sampling in East Asia, and the fact that high genetic diversity was observed in Southeast Asia, which is not consistent with a recent migration of the species into this region. Moreover, a sampling gap existed in Vietnam and in western part of China, which may have biased the interpretation of the results. Their study could unambiguously discard Taiwan as a potential source area. On the other hand, a more recent study [20] focused on China and was based on mitochondrial sequences only. This work suggested that genetic diversity is very high in *B. dorsalis*, and that genetic structure is weak, even at this geographical scale. The authors [20] could nonetheless identify three population groups, and suggested that the species could be native along the coast of the China Sea and expanded recently in northern regions. Expansion patterns were supposed to be gradual, as no sign of reduction of genetic diversity were found. In this case [20], the conclusions could suffer from the lack of sampling in Southeast Asian countries, where Aketarawong et al. [7] found a source of diversity, and from the use of a single, maternally-inherited marker.

The present study is based on an extensive geographical sampling of the oriental fruit fly, as it includes most regions studied by Akeratawong et al. [7] and Wan et al. [20], in particular those regions previously identified as potential source areas. We then applied both multilocus microsatellite loci and mitochondrial DNA sequencing, to avoid any bias due to the use of one type of marker only [11], [21]. Microsatellites are nuclear, codominant loci, with high levels of variability; they are particularly informative to study recent population processes [11]. By contrast, maternally inherited mitochondrial markers can provide a deeper understanding of invasion history and evolutionary processes [11], [22]. We used these makers to infer the genetic structure and analyze the distribution of genetic diversity of *B. dorsalis* over a large geographical scale in China and Southeast Asia. We also aimed to identify the native region of this pest species and its colonization pathways in Asia.

## Materials and Methods

### Studied system and historical data

Phytophagous flies of the Tephritidae family, also called “true fruit flies”, are among the most important pests of fruits and vegetables. One-third of the species in this large family (more than 4000 known species) attack soft fruits, including many commercially and economically important species [23]. Tephritid flies such as *Bactrocera oleae* (Gmelin), *Bactrocera cucurbitae* (Coquillett), or *Ceratitis capitata* (Wiedemann) represent cases of successful invasive species, which have caused tremendous economic losses in many countries [5], [24] and are thus ranked among the most important quarantine insects worldwide [23]. Global warming, regional trade, tourism are factors favoring the dispersion of Tephritidae pests [24]. For example, *Bactrocera invadens*, a recently described species of Tephritidae, has expanded throughout Africa within only two years after its first discovery and is currently causing extensive agricultural losses [23].

*B. dorsalis* typically occurs in tropical areas of South, East, and Southeast Asia (including Bhutan, south of mainland China, Hong-Kong, Taiwan, India, Japan, Laos, Myanmar, Nepal, Bangladesh, Sri-Lanka, Vietnam, Cambodia, Pakistan and Thailand) as well as in some Pacific islands (e.g., Hawaii, Guam, Northern Mariana, Nauru and French Polynesia) [25]. In China, *B. dorsalis* was first reported in 1934 in Hainan province [26] after which it was never reported again in China for the next 20 years. Then, after 1950, *B. dorsalis* infestations were reported in southwestern and southeastern parts of China [27]. *B. dorsalis* is now distributed mainly in provinces located south of the Yangtze River in mainland China [28] (including Guangxi, Guangdong, Fujian, Yunnan, Guizhou, Sichuan, Hunan and Hainan) and in Taiwan [29]. Conversely, the regions situated north of the Yangtze River are thought to be unsuitable for this fly as suggested by results of CLIMEX modeling [30].

### Ethics statement

No specific permits were required in our studies of this widespread agriculture pest. We confirm that the study locations were not privately owned or protected. This work did not involve endangered or protected species.

### Sampling and DNA extraction

**Figure 1:**
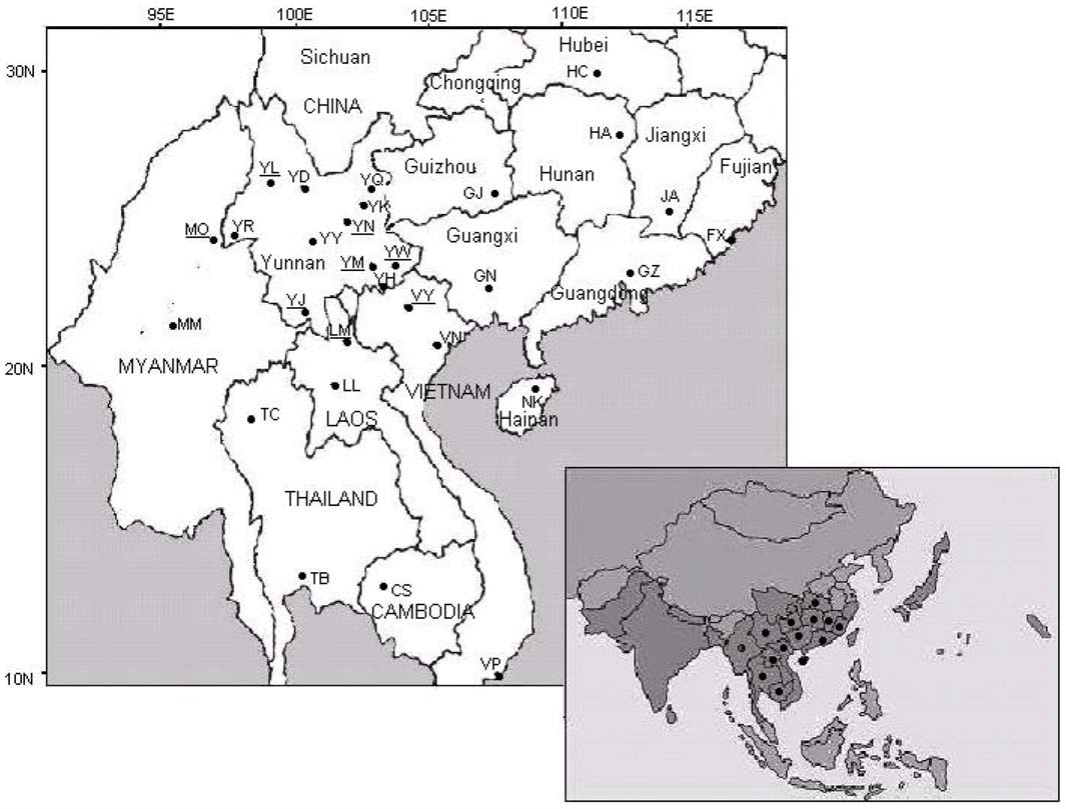
Sampling sites of *B. dorsalis*, coded according to **Table 1**. Sites that are underlined correspond to samples for which only mtDNA was studied. The map in the lower right corner is the known distribution range of *B. dorsalis* in Asia (in dark grey), and • represents the countries and provinces where we sampled *B. dorsalis* during the present study.

*B. dorsalis* adults were collected from 21 localities in China and Southeast Asian countries. In China, we sampled 9 provinces, namely Yunnan, Guizhou, Guangxi, Fujian, Guangdong, Hainan, Jiangxi, Hubei and Hunan. From Southeast Asia we sampled in Myanmar, Vietnam, Laos, Thailand and Cambodia. Details about sampling sites and sampling sizes are given in Table 1 and the localities are shown in Fig. 1. All the samples were obtained as ethanol preserved adults and DNA was extracted from each single fly according to the method of Shi et al. [17]. Moreover, we merged mitochondrial data obtained from the present study with previous results [18], thereby obtaining a dataset for a total of 29 populations (see Table 1; Fig. 1). The microsatellite data were only obtained for the 21 newly sampled sites.

**Table 1:** Sampling details for 29 *B. dorsalis* populations used in this study.

Country	Sample name	Sample site	Code	Date of collect	Coordinates	Host Plant	Molecular markers	Sample size
Myanmar	Myanmar-M	Mandala	MM	Jun-09	21°58′N, 96°04′E	Mango	Microsatellites and mitochondrial DNA	20
	Myanmar-O	Bhamo	MO	Aug-06	24°16′N, 97°17′E	Mango	Mitochondrial DNA	20
Vietnam	Vietnam -P	Panchit	VP	Jul-09	10°56′N, 108°06′E	Mango	Microsatellites and mitochondrial DNA	20
	Vietnam -N	Hanoi	VN	Jul-09	21°02′N, 105°51′E	Mango	Microsatellites and mitochondrial DNA	20
	Vietnam-Y	Yên Bái	VY	Aug-06	21°70′N, 104°86′E	Mango	Mitochondrial DNA	20
Laos	Laos-L	Luang Prabang	LL	Jul-09	19°53′N, 102°09′E	Mango	Microsatellites and mitochondrial DNA	20
	Laos-M	Muang Khua	LM	Aug-06	21°08′N, 102°50′E	Mango	Mitochondrial DNA	20
Cambodia	Cambodia	Siem Reap	CS	Jul-09	13°21′N, 103°51′E	Mango	Microsatellites and mitochondrial DNA	20
Thailand	Thailand-C	Chiang Mai	TC	May-10	18°47′N, 98°59′E	Mango	Microsatellites and mitochondrial DNA	20
	Thailand-B	Bangkok	TB	May-10	13°45′N, 100° 30′E	Mango	Microsatellites and mitochondrial DNA	20
China	Yunnan-Y	Yuanjiang	YY	Aug-05	23°36′N, 101°59′E	Mango	Microsatellites and mitochondrial DNA	20
	Yunnan-K	Kunming	YK	Aug-05	25°01′N, 102°41′E	Apple	Microsatellites and mitochondrial DNA	20
	Yunnan-D	Dali	YD	Aug-05	25°42′N, 100°72′E	Apple	Microsatellites and mitochondrial DNA	20
	Yunnan-Q	Qujing	YQ	Aug-05	25°30′N, 103°48′E	Apple	Microsatellites and mitochondrial DNA	20
	Yunnan-R	Ruili	YR	Aug-06	24°01′N, 97°51′E	Mango	Microsatellites and mitochondrial DNA	20
	Yunnan-H	Hekou	YH	Aug-05	22°30′N, 103°57′E	Mango	Microsatellites and mitochondrial DNA	20
	Yunnan-N	Huanian	YN	Aug-06	24°03′N, 102°12′E	Mango	Mitochondrial DNA	20
	Yunnan-J	Jinghong	YJ	Aug-05	21°59′N, 100°48′E	Mango	Mitochondrial DNA	20
	Yunnan-W	Wenshan	YW	Aug-06	23°23′N, 104°15′E	Mango	Mitochondrial DNA	20
	Yunnan-M	Menzi	YM	Aug-06	23°23′N, 103°23′E	Mango	Mitochondrial DNA	20
	Yunnan-L	Liuku	YL	Aug-06	25°52′N, 98°51′E	Mango	Mitochondrial DNA	20
	Guizhou	Rongjiang	GJ	Jul-09	25°55′N,108°31′E	Pear	Microsatellites and mitochondrial DNA	20
	Guangxi	Nanning	GN	Jul-09	22°47′N, 108°21′E	Mango	Microsatellites and mitochondrial DNA	20
	Guangdong	Guangzhou	GZ	Aug-09	23°13′N, 113° 27′E	Mango	Microsatellites and mitochondrial DNA	20
	Fujian	Xiameng	FX	Aug-09	24°04′N, 118°01′E	Mango	Microsatellites and mitochondrial DNA	20
	Hainan	Haikou	NK	Aug-09	18°25′N, 109°50′E	Mango	Microsatellites and mitochondrial DNA	20
	Jiangxi	Anyuan	JA	Aug-09	25°89′N, 114°90′E	Mango	Microsatellites and mitochondrial DNA	20
	Hubei	Changyang	HC	Aug-09	32°08′N, 112°02′E	Apple	Microsatellites and mitochondrial DNA	20
	Hunan	Aanhua	HA	Aug-09	27°72′N, 113°13′E	Mango	Microsatellites and mitochondrial DNA	20

### Laboratory procedures

#### Mitochondrial sequencing

A 601 bp fragment of the mitochondrial cytochrome oxidase gene (COI) was amplified and sequenced using the protocols described in Shi et al. [17]. PCR products were gel purified (Watson Biotechnologies, Shanghai, China) and sequenced in both directions in an ABI 377 automated sequencer (Applied Biosystems, Warrington, UK). As nuclear copies of mitochondrial genes (numts) can generate doubtful results [31], [32], we systematically double-checked the obtained chromatograms to ensure that double peaks did not occur and that the haplotypes were functional coding genes (absence of indels or stop codons). The DNA sequences were edited manually and aligned using ClustalX as implemented in BIOEDIT 7.0 [33]. After manual correction and assembly, unique sequences were deposited in GenBank with accession numbers JQ037859–JQ037889.

#### Microsatellite genotyping

Seven microsatellite loci were used in this study. Technical details are given in Dai et al. [34] for the five loci MS3, MS3B, MS4, MS5, MS12A, and in Li et al. [35] for loci 618A and BO-D48. Electrophoresis was carried out using an automated ABI PRISM 310 Genetic Analyzer and allele calling was performed using GeneMapper. An individual was declared null for a given locus only after at least two amplification failures. Microsatellite data were deposited in the Dryad Repository: doi:10.5061/dryad.5qg356qk

### Within-population genetic diversity indices

#### Mitochondrial data

Numbers and distribution of haplotypes, numbers of unique haplotypes, polymorphic sites, within-population mean number of pairwise differences between pairs of haplotypes (π) and nucleotide diversity [36] were assessed using ARLEQUIN 3.11 [37].

#### Microsatellite data

For each population, the following genetic diversity estimates were calculated as averages over all microsatellite loci with GENEPOP 4.0 [38]: mean number of alleles (*n*_a_); number of private alleles (*n*_p_); frequency of private alleles (*A*_P_); observed heterozygosity (*H*_O_) and expected heterozygosity (*H*_E_). Gene diversity (*H*_S_) and allelic richness (*R*_S_) were calculated using FSTAT 2.9.3.2 [39]. GENEPOP 4.0 was also used to test for linkage disequilibrium between pairs of loci in each population and for deviation from Hardy-Weinberg equilibrium (HWE) based on Fisher's method, after the sequential Bonferroni correction [40]. The frequency of null alleles was estimated using FreeNA [41].

### Population genetic structure

#### Pairwise F_ST_

The degree of genetic differentiation between pairs of populations was measured by pairwise F_ST_ estimates for both types of markers. They were calculated using ARLEQUIN for mitochondrial sequences, and estimated using the ENA (Excluding Null Alleles) correction described in Chapuis & Estoup [41] for microsatellite data using FreeNA. The statistical significance of each value was assessed by the comparison of the observed value with the values obtained in 10,000 permutations.

#### Isolation by distance (IbD)

To detect isolation by distance, matrices of pairwise estimates of genetic differentiation (F_ST_ values) were compared with the matrix of geographic distances by means of a simple Mantel test [42]. The Mantel test quantifies the correlation between two distance matrices, therefore allowing determination of a relationship between genetic and geographical distances. It was performed using ARLEQUIN for mitochondrial data, and using the program ISOLDE within GENEPOP 4.0 for microsatellites.

#### Phylogenetic analyses

Genealogical relationships between mitochondrial haplotypes were reconstructed using TCS 1.21 [43] with the method described by Templeton et al. [44]. POPULATIONS 1.2.30 [45] was used to construct unrooted neighbor-joining (NJ) trees based on pairwise proportion-of-shared-alleles distances (*D*_s_) calculated with microsatellite data [46]. Support for tree nodes was assessed by 1000 bootstrap resamplings of the original data set over loci. The same program was used to construct the NJ tree based on the mitochondrial genetic distance matrix between 29 populations.

#### Assessment of population groups

Spatial analysis of molecular variance (SAMOVA) was performed with both types of markers separately using SAMOVA 1.0 [47] to identify groups of populations that are phylogeographically homogeneous and maximally differentiated from each other, taking into account the geographic distances. This analysis permits identification of the maximally differentiated groups that correspond to predefined genetic barriers by maximizing the proportion of total genetic variance due to differences between groups [48]. To select the optimal number of groups (K), two criteria must be considered. First, F_CT_ values should reach a maximum or a plateau. Second, the configurations with one or more single-population groups should be excluded because this indicates that the group structure is disappearing [49]. We performed analyses for K = 2 to 10 to identify the most likely number of groups, with the 21 populations genotyped with microsatellites and 29 populations characterized by mitochondrial sequences. Analyses of molecular variance (AMOVA) [50] were then performed to test the genetic relationships between the different groups defined by SAMOVA.

For microsatellite markers, the Bayesian approach implemented in STRUCTURE 2.3.3 [51] was further used to infer the clustering of the 21 studied populations. The program STRUCTURE uses the individual as the unit, assessing whether it belongs to one or more population groups or clusters, regardless of its geographical origin. It assumes a model in which there are K clusters (K being unknown), each of which is characterized by a set of allele frequencies at each locus. Individuals in the samples are probabilistically assigned to one cluster, or jointly to two or more clusters if their genotypes indicate that they are admixed. The optimal number of clusters (K) represented by the data can be found by comparing the estimated log probability of the data for the different values of K [51]. We used 100,000 burn-in steps followed by 100,000 MCMC simulation steps with a model allowing admixture. To assess the consistency of results, we performed 10 independent runs for each value of K (from 2 to 13) and carefully compared the obtained individual Q-matrices.

#### Potential effect of host plant

AMOVAs were run on the mitochondrial data set as well as on the microsatellite data to test the effect of the host plants on the genetic structure of the fly. Flies were thus grouped depending on the host they were sampled from (mango, apple and pear). Such analyses were performed using ARLEQUIN.

### Demographic history

#### Mitochondrial data

To study the demographic history of the groups of populations identified previously, we studied mismatch distributions [52] and we calculated Tajima's *D* and Fu's *F_S_* for the 8 identified SAMOVA groups. We then tested whether these indices significantly deviated from 0. All demographic analyses were performed using ARLEQUIN.

#### Microsatellite data

The program BOTTLENECK 1.2.02 [53] was used to detect an eventual recent bottleneck and expansion in each population. One of the assumptions of this method is that allele frequency distribution results from an equilibrium between mutation and genetic drift [54]. Recent bottlenecks provoke a shift away from an L-shaped distribution of allelic frequencies, to one with fewer alleles at low frequency categories. Under specific assumptions relative to the mutation model, the methods imply that shrinking populations reduce allelic diversity faster than heterozygosity or gene diversity [55]. An excess of observed gene diversity relative to the expected gene diversity for the number of alleles detected in the sample may indicate a population size reduction. Conversely, a deficit in the observed gene diversity may indicate that the population is growing. Two mutation models, considered appropriate for microsatellites [53], were applied: the strict Stepwise Mutation Model (SMM) and the Two-Phase Model (TPM). For the TPM, a model that includes both 90% single-step mutations and 10% multiple step mutations was used. Significant deviations in observed heterozygosity over all loci were tested using a non parametric Wilcoxon test. The same software was used to estimate recent bottleneck or expansion for the 7 microsatellite SAMOVA groups.

### Migration rate estimates

#### Mitochondrial data

The coalescent-based strategy implemented in MIGRATE 3.2.17 [56] was used to test the extent of gene flow between population pairs. This strategy tests the existence of asymmetrical gene flow between populations. The mutation scaled effective immigration rate (M = m/μ) ingoing and outgoing per population and per generation was estimated applying the Bayesian search strategy. We set one long chain of 100,000,000 generations with the initial 10,000 excluded as burn-in.

#### Microsatellite data

The GENECLASS 2.0 software [57] was used to assign or exclude reference populations as possible origins of individuals on the basis of multilocus genotypes. The program calculates, for each individual, the probability that it actually belongs to any other reference population or that it is a resident in the population where it was sampled. We used the standard criterion described by Rannala & Mountain [58], which applies Bayesian statistics to compute probabilities. The Monte Carlo resampling method [59] was applied to identify the accurate exclusion/inclusion critical values; our results were based on 10,000 simulated genotypes for each population and on a threshold probability value of 0.01.

## Results

### Markers characteristics and within-population genetic diversity indices

#### Mitochondrial data

Partial sequences of the mitochondrial COI gene were obtained for 420 individuals of *B. dorsalis* from 21 populations. We merged these sequences with previously published sequences (accession numbers DQ06028–060304, DQ100468, DQ100470–100471, GQ414975–414988) from five populations in Yunnan province (YN, YJ, YW, YM and YL) and three Southeast Asian populations (MO, VY and LM) [17], [18] to obtain a final alignment for 580 *B. dorsalis* flies from 29 localities. The total alignment was 519 base pairs (bp) long. There were 85 polymorphic sites and 73 haplotypes were observed (see Table 2), of which 31 were found in this study and 42 were already identified in previous work [17], [18].

**Table 2:** Genetic diversity indices based on mitochondrial data.

Country	Population	Nucleotide diversity	Average number of pairwise differences within populations	Number of private haplotypes	Number of haplotypes	Haplotype distribution
Myanmar	MM	0.020	9.11	3	10	H1(1),H2(2), H3(2), H4(3),H5(3), H6(2), H7(2), H8(2),H9(2) H10(1)
	MO	0.014	8.01	2	9	H1(1), H6(3), H7(4),H8(1),H9(2), H11(4), H12(1), H13(2), H14(2)
Vietnam	VP	0.017	8.92	3	11	H15(1),H16(2),H17(2),H18(1),H19(2),H20(1),H21(2),H22(2),H23(2),H24(2),H25(3)
	VN	0.013	8.20	0	9	H17(3), H18(2), H19(4),H21(2), H22(2), H23(2), H24(2), H25(2), H26(1)
	VY	0.015	7.30	1	7	H17(2), H18(3), H23(3), H26(3), H27(2), H28(4), H29(3)
Laos	LL	0.015	7.14	0	9	H30(5),H31(4), H32(3), H33(2), H34(1), H35(2), H36(1), H37(1), H38(1)
	LM	0.012	6.46	0	6	H32(4), H33(3), H37(2), H38(2), H39(5),H40(4)
Cambodia	CS	0.016	8.14	0	6	H23(1), H41(1),H42(1),H43(8),H44(4), H45(5)
Thailand	TC	0.017	7.83	0	5	H39(4), H41(4), H42(4), H44(4), H45(4)
	TB	0.020	8.29	1	6	H39(3), H41(4), H42(3),H43(3), H45(4), H46(3)
China	YY	0.016	6. 30	0	6	H47(3), H48(3),H49(4),H50(4),H51(3), H52(3)
	YK	0.012	6.15	0	8	H10(3), H47(3), H48(1), H50(2), H51(2),H52(2),H53(5), H54(2)
	YD	0.009	4.97	0	5	H48(1), H49(2), H50(13), H51(2), H54(2)
	YQ	0.011	5.85	0	4	H50(3), H51(9), H52(4), H54(4)
	YR	0.012	6.38	0	8	H5(2), H6(3), H7(2), H10(5), H11(1), H12(3), H13(3), H55(1)
	YH	0.008	4.30	0	7	H23(4), H24(3), H25(2), H26(2), H27(3),H28(3), H56(3)
	YN	0.013	6.85	0	9	H10(1), H33(2), H47(2),H48(2), H50(2), H51(2), H52(2), H53(3), H54(4)
	YJ	0.013	6.73	1	8	H32(2), H33(2), H34(4), H35(3), H36(3), H39(2), H40(3), H57(1)
	YW	0.007	3.41	0	5	H23(3), H26(4), H27(5), H28(4), H56(4)
	YM	0.010	5.59	1	4	H26(6), H27(5), H56(5), H58(4)
	YL	0.008	3.98	0	4	H10(10), H11(4), H13(3), H55(3)
	GJ	0.014	4.16	0	4	H10(3), H19(7), H22(4), H25(6)
	GN	0.008	6.04	0	5	H19(4), H22(3), H25(6), H61(5), H62(2)
	GZ	0.015	7.46	3	9	H19(1), H25(2), H62(3), H63(3), H64(2), H65(3), H66(1), H67(3), H68(2)
	FX	0.010	6.86	2	6	H65(3), H69(2), H70(4), H71(4), H72(4), H73(3)
	NK	0.011	5.65	0	6	H19(3), H25(3), H62(4), H65(3), H66(4), H67(3)
	JA	0.013	5.62	0	4	H65(4), H66(6), H70(5), H72(5)
	HC	0.009	5.21	0	4	H59(4), H60(6), H65(5), H70(5)
	HA	0.012	5.38	0	4	H59(5), H60(5), H70(4), H71(6)

Average number of pairwise differences between all pairs of haplotypes within populations (π), nucleotide diversity, number of private haplotypes, number of haplotypes and haplotype distribution for each sampled population of *B. dorsalis*, numbers in brackets correspond to the number of individuals with this haplotype.

Four types of indices including nucleotide diversity, average number of pairwise differences within populations (π), number of haplotypes and number of unique haplotypes were calculated to measure genetic diversity within population (see Table 2). Among the 29 fly populations, all four indices were maximal in VP (nucleotide diversity = 0.017, π = 8.92, haplotype number = 11, unique haplotype = 3) and MM (nucleotide diversity = 0.020, π = 9.11, haplotype number = 10, unique haplotype = 3). The four indices were also high in VN, CS and TB. Among all the *B. dorsalis* populations analyzed from China, all the genetic diversity indices were high in GZ while the four indices were lowest in YW.

#### Microsatellite data

Our data were based on 420 *B. dorsalis* flies sampled across 21 populations and genotyped at 7 microsatellite loci. Number of alleles per locus ranged from 12 to 27. After the sequential Bonferroni correction [40], the majority of the sampled populations conformed to HWE at most of the loci. The locus/population combinations that were not in HWE were not concentrated at one locus or in one population. The average frequencies of null alleles per locus were 0.039 for locus 618A, 0.038 for Bo-D48, 0.065 for MS3, 0.082 for MS3B, 0.069 for MS4, 0.071 for MS5, and 0.069 for MS12A. Overall, mean frequency of null alleles for each locus was always below 0.10. No linkage disequilibrium was observed for any pair of loci.

Table 3 lists the genetic variability indices estimated over the 7 microsatellite loci for each population. Consistent with mitochondrial data, the sites VP and MM presented the highest indices of genetic diversity including number of alleles, private alleles with high frequency (>0.30), expected heterozygosity among the 21 studied populations, and the sites VN, CS and TB also showed relatively high within-population indices of diversity. On the other hand, the YD population had the lowest indices of genetic variation with very low frequency. Within China, GZ and FX presented the highest levels of genetic variation and the frequency of private allele was also high (>0.25).

**Table 3:** Genetic variability estimates for 21 *B. dorsalis* populations based on microsatellite data.

Population	*n*_a_	*n*_p_	*A*_p_	*R*_S_	*H*_S_	*H*_O_	*H*_E_
MM	6.86	8	0.035	6.86	0.732	0.593	0.729
VP	8.71	10	0.040	8.71	0.724	0.600	0.721
VN	7.14	7	0.030	7.14	0.665	0.556	0.663
LL	4.86	0	0.000	4.86	0.569	0.586	0.569
CS	5.71	6	0.025	5.71	0.658	0.686	0.659
TC	7.14	2	0.065	7.14	0.675	0.536	0.671
TB	7.57	6	0.038	7.57	0.675	0.607	0.673
YY	4.57	3	0.073	4.57	0.596	0.627	0.599
YK	4.51	0	0.000	4.51	0.553	0.607	0.554
YD	3.46	0	0.000	3.46	0.544	0.629	0.547
YQ	4.42	1	0.030	4.42	0.534	0.586	0.556
YR	5.02	0	0.000	5	0.582	0.536	0.580
YH	3.71	2	0.110	3.71	0.552	0.643	0.554
GJ	4.71	0	0.000	4.71	0.661	0.600	0.662
GN	5.71	1	0.050	5.71	0.679	0.579	0.677
GZ	6.43	4	0.026	6.43	0.685	0.586	0.682
FX	5.43	4	0.030	5.43	0.685	0.586	0.683
NK	4.71	1	0.050	4.71	0.620	0.507	0.617
JA	5.24	0	0.000	5.24	0.639	0.536	0.645
HC	4.28	1	0.150	4.28	0.624	0.521	0.616
HA	5.57	0	0.000	5.57	0.649	0.520	0.644

*n*_a_: mean number of alleles; *n*_p_: number of private alleles; *A*_p_: mean frequency of private alleles; *R*_s_: allelic richness; *H*_s_: gene diversity; *H*_O_: mean observed heterozygosity; *H*_E_: mean expected heterozygosity.

### Population genetic structure

#### Pairwise F_ST_

Pairwise F_ST_ estimates were used to measure the genetic structure of the 29 *B. dorsalis* populations using mitochondrial sequences, and 21 populations genotyped with microsatellites. Table S1 lists all mitochondrial pairwise F_ST_ values that ranged from 0.01 (YK/YY) to 0.4 (YL/YQ). Concerning microsatellite data, Table S1 shows the pairwise F_ST_ values after ENA correction, that ranged from 0.01 (TB/TC and FX/VP) to 0.22 (HC/YQ). Most values were significant.

#### Isolation by distance

The Mantel tests performed with both kinds of markers showed that the correlations between geographic distances and pairwise F_ST_ were not significant (MtDNA: standardized Mantel statistics r = 0.179, P = 0.235; microsatellites: standardized Mantel statistics r = 0.276, P = 0.055), indicating the absence of IbD.

#### Phylogenetic analyses

Fig. S1 shows the 95% parsimony network obtained for the 73 mitochondrial haplotypes. 98 missing haplotypes were detected. The network lacked clear structure and no phylogenetic haplogroup could be identified. Moreover, the haplotypes present in any given locality or region were not phylogenetically related, but scattered over the whole network. Yet, groups of unrelated haplotypes were shared between geographically close populations, and restricted to that group of sampling sites (see SAMOVA results below).

**Figure 2:**
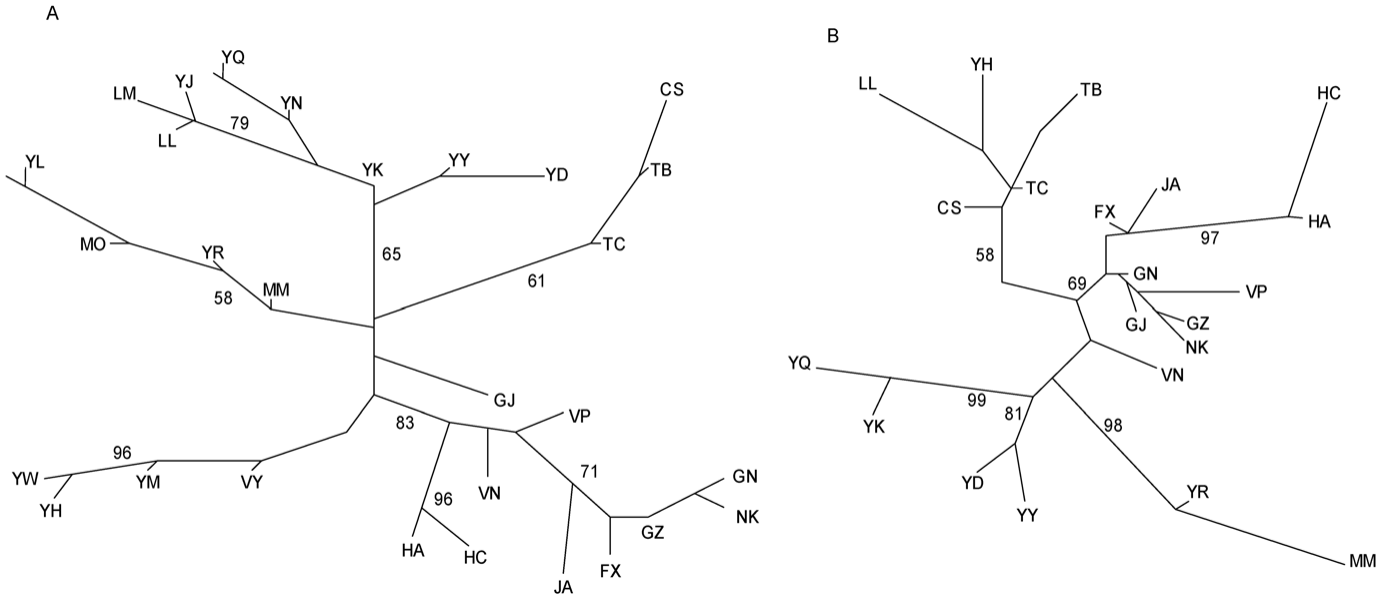
Neighbor-joining unrooted trees based on both molecular markers. A: unrooted tree based on mitochondrial genetic distances (F_ST_) matrix. B: unrooted tree based on the proportion of shared alleles for microsatellite data. Numbers at each node indicated the bootstrap values after 1000 replicates. Only values above 50% are shown.

An unrooted NJ tree (Fig. 2A) was constructed based on mitochondrial genetic distances. Five population groups can be identified, while population GJ was somewhat isolated. A first group was formed by (YW, YH, YM and VY), a second one by (MO, MM, YR and YL), a third one by (CS, TB and TC). The two other population groups found in the phylogenetic tree contained 8 and 9 localities, namely (GN, NK, GZ, FX, JA, HC, HA, VN, VP) which was very close to GJ, and (LM, LL, YJ, YQ, YN, YK, YY and YD).

The NJ tree of populations constructed with the microsatellite data (based on the pairwise proportion-of-shared–alleles distances) also showed five monophyletic clades (Fig. 2B). Three pairs of populations (YD+YY, YQ+YK, YR+MM) formed three differentiated groups that were close to each other. Populations GN, VP, GZ, NK, GJ, VN, HC, HA, JA and FX formed a large monophyletic group. The remaining populations (LL, YH, TC, TB and CS) formed the last monophyletic group. The identified clades showed a strong geographical pattern.

#### Assessment of population groups

**Figure 3:**
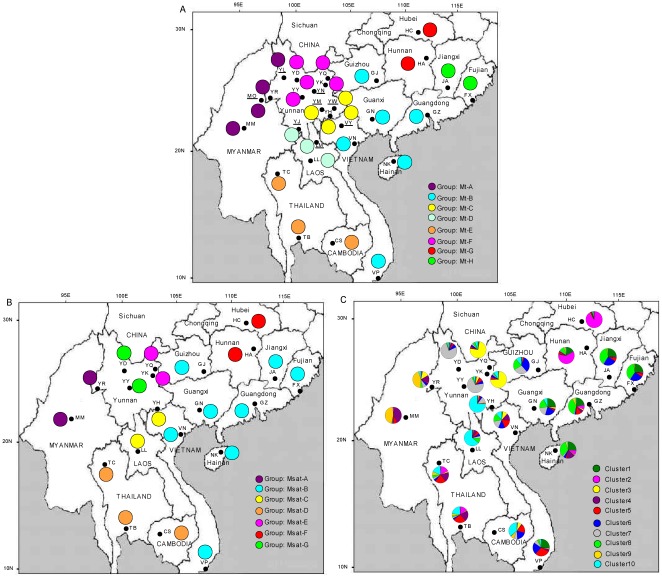
Group structure for *B. dorsalis* populations based on two molecular markers. A: Color codes of populations correspond to the 8 groups identified by SAMOVA inferred from mitochondrial sequences (29 *B. dorsalis* populations). Colors are the same as in Fig. 2. B: Color codes of populations correspond to the 7 groups identified by SAMOVA inferred from microsatellite data (21 populations). C: Color codes of populations represents the relative frequency of each of the 10 clusters identified using the Structure software with microsatellite results.

SAMOVA was performed to identify genetic groups of populations, using either mitochondrial or microsatellite data. For the first data set, the F_CT_ value was highest for K = 8 (Fig. S2 A) and no single-population group was formed. SAMOVA thus identified 8 groups of populations based on mitochondrial data, that we named Mt-A, Mt-B, Mt-C, Mt-D, Mt-E, Mt-F, Mt-G and Mt-H, respectively (see Table 4). The 8 groups were geographically consistent and corresponded to regions (Fig. 3A). The AMOVA run using this particular grouping of populations revealed that most of the molecular variance was found within populations (82.12%, P<0.001). Yet, 12.86% of total variance was found among different groups, and this partition was significant (P<0.001). When run with the microsatellite data, the SAMOVA results were very similar to those obtained with mitochondrial sequences, except that the most plausible number of population groups was 7 (Fig. S2 B, Table 4: groups Msat-A, Msat-B, Msat-C, Msat-D, Msat-E, Msat-F and Msat-G). The regional pattern was very similar to the results obtained from mitochondrial DNA (Fig. 3B). The corresponding AMOVA showed that 86.07% of molecular variance was found within populations and 10% among groups, both values being significant (P<0.001). In general, the patterns found with the SAMOVA analyses were consistent with the phylogenetic trees of populations.

**Table 4:** SAMOVA results based on mitochondrial and microsatellite markers.

	mtDNA						Microsatellites				
Group name	Groups	*D*	*Fs*	Source of variation	% of variation	Fixation indices	Group name	Groups	Source of variation	% of variation	Fixation indices
Mt-A	MM+MO+YR+YL	0.39	−8.76				Msat-A	MM+YR			
Mt-B	VP+VN+GN+GJ+GZ+NK	−0.6	−4.75	Among groups	12.86	F_CT_ = 0.1286**	Msat-B	VP+VN+GN+GJ+GZ+FX+JA+NK	Among groups	10.04	F_CT_ = 0.1004**
Mt-C	VY+YW+YM+YH	−2.47**	−23.56**				Msat-C	YH+LL			
Mt-D	LL+LM+YJ	1.4	−3.55	Among populations within groups	5.02	F_SC_ = 0.059**	Msat-D	CS+TC+TB	Among populations within groups	3.89	F_SC_ = 0.042**
Mt-E	CS+TC+TB	1.17	1.33				Msat-E	YK+YQ			
Mt-F	YY+YK+YD+YQ+YN	−1.92**	−17.79**				Msat-F	HC+HA			
Mt-G	HC+HA	−2.43**	−24.34**	Within populations	82.12	F_ST_ = 0.199**	Msat-G	YY+YD	Within populations	86.07	F_ST_ = 0.119**
Mt-H	FX+JA	1.13	2.07								

*D*: Tajiama's *D*; *Fs* : Fu's *Fs* are given for the SAMOVA groups based on mitochondrial data.

** P<0.001.

We also analyzed the population genetic structure based on microsatellites using the STRUCTURE software. The analysis indicated that the 21 *B. dorsalis* populations could be subdivided into 10 different hypothetical genetic clusters (K), as shown by the likelihood curve (Fig. S2 C, 10 runs for each K), as the likelihood estimate reached a plateau for K = 10. Each of the 420 flies was subsequently assigned, entirely or in part, to each of the 10 clusters with a certain probability Q (Table 5, Fig. 3C). Concerning Southeast Asia, flies from MM were mostly assigned to cluster 9 (Q = 0.455) and cluster 4 (Q = 0.386). The ancestry of flies from VP, VN, CS, TC and TB was fragmented and individuals were admixed in several clusters, with Q<0.36 for all of them. Population LL was mostly assigned to cluster 10 (Q = 0.641). In China, 7 of the 14 sampling sites were mostly assigned to one cluster with high Q values (namely HC and HA to cluster 2, YK and YQ to cluster 3, YY and YD to cluster 7 and YH to cluster 10). The seven remaining sites (YR, GJ, GN, FX, JA, GZ and NK) had moderate population Q-values, with admixed individuals well assigned to different clusters. The pattern obtained is globally consistent with the SAMOVA results, but the signal is blurred by high levels of admixture (Fig. 3C).

**Table 5:** Estimated membership probabilities (Q) of 21 *B. dorsalis* populations into 10 hypothetical ancestry clusters inferred by STRUCTURE.

	Cluster									
Population	1	2	3	4	5	6	7	8	9	10
MM	0.006	0.025	0.006	0.386	0.076	0.015	0.008	0.014	**0.455**	0.008
VP	*0.262*	0.008	0.016	0.044	**0.291**	*0.224*	0.074	0.034	0.016	0.032
VN	0.019	0.007	0.097	*0.136*	**0.174**	*0.133*	*0.156*	*0.147*	0.007	*0.123*
LL	0.009	0.011	0.009	0.079	0.058	0.046	0.046	0.096	0.005	**0.641**
CS	0.008	0.011	0.081	0.022	0.167	*0.204*	0.072	0.068	0.007	**0.361**
TC	0.007	*0.172*	0.022	*0.193*	**0.216**	0.043	0.038	0.053	0.065	*0.192*
TB	0.007	*0.146*	0.010	*0.217*	**0.266**	0.033	0.025	0.027	0.045	*0.224*
YY	0.014	0.010	0.062	0.012	0.070	0.060	**0.697**	0.027	0.006	0.041
YK	0.006	0.007	**0.732**	0.011	0.044	0.051	0.025	0.019	0.004	0.101
YD	0.014	0.008	0.011	0.039	0.035	0.076	**0.759**	0.013	0.006	0.037
YQ	0.023	0.007	**0.745**	0.010	0.028	0.043	0.028	0.044	0.005	0.067
YR	0.012	0.025	*0.129*	*0.142*	0.029	0.051	0.079	0.037	**0.441**	0.054
YH	0.015	0.007	0.016	0.012	0.020	0.038	*0.117*	0.017	0.007	**0.751**
GJ	0.010	0.009	0.012	0.011	0.023	**0.317**	*0.282*	*0.217*	0.005	0.115
GN	**0.249**	0.013	0.030	0.019	0.027	*0.206*	*0.181*	*0.191*	0.005	0.079
GZ	*0.233*	0.083	0.024	0.059	*0.105*	0.028	0.019	**0.364**	0.060	0.024
FX	*0.247*	0.013	0.019	0.017	0.054	**0.318**	0.045	*0.245*	0.006	0.036
NK	*0.255*	0.091	0.014	*0.160*	0.031	0.046	0.032	**0.316**	0.033	0.023
JA	*0.243*	0.011	0.008	0.013	0.064	*0.252*	0.015	**0.368**	0.017	0.008
HC	0.005	**0.899**	0.011	0.010	0.023	0.007	0.009	0.019	0.010	0.007
HA	*0.172*	**0.586**	0.008	0.022	0.038	0.011	0.015	0.096	0.032	0.019

The highest value of co-ancestry of each population is in bold. Values higher than 0.10 are italics.

#### Potential effect of host plants

To test the effect of host plant species on the genetic structure of *B. dorsalis*, the sampled populations were split into three groups according to the host-plant (mango, apple and pear) and subjected to AMOVA. The results based either on mitochondrial or on microsatellite data showed that no significant differences were found among groups (see Table 6). Most molecular variance was always found within populations.

**Table 6:** AMOVA test for different host groups for *B. dorsalis* populations based on two molecular markers.

	mtDNA				Microsatellites			
Group name	Group competent	Source of variation	% variation	Fixation indices	Group competent	Source of variation	% variation	Fixation indices
Mango	MM+MO+VP+VN+VY+LL+LM+CS+TC+TB+YY+YR+YH+YW+YM+YN+YL+YJ+GN+FX+HA+JA+GZ+NK	Among group	1.56	F_CT_ = 0.0156	MM+VP+VN+LL+CS+TC+TB+YY+YR+YH+GN+FX+HA+JA+GZ+NK	Among group	1.65	F_CT_ = 0.0165
Apple	YK+YQ+YD+HC	Among pops within groups	19.22	F_SC_ = 0.1892**	YK+YQ+YD+HC	Among pops within groups	10.07	F_SC_ = 0.1024**
Pear	GJ	Within pops	82.34	F_ST_ = 0.177**	GJ	Within pops	88.27	F_ST_ = 0.1173**

** **P<0.001.**

### Demographic history

**Figure 4:**
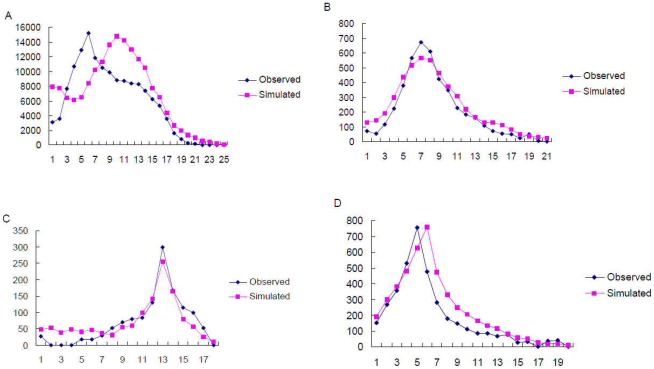
Analyses of mismatch distributions. A, B, C, D show mismatch distributions for the complete dataset, and the SAMOVA groups Mt-C, Mt-G and Mt-F, respectively. The horizontal axis represents the number of pairwise differences; the vertical axis represents the relative frequency.

Neutrality tests (Tajima's *D* and Fu's *Fs*) were performed on mitochondrial data for the whole data set and for each of the 8 groups identified by SAMOVA. Significant negative values were found in the whole dataset (Tajima's *D* = −0.916, P*_D_* = 0.0173; *Fs* = −23.99, P*_Fs_* = 0.005) and three of the SAMOVA groups, namely Mt-C, Mt-G and Mt-F (Table 4), indicating that the whole set of *B. dorsalis* and populations of these three groups, from Western and Northern China, did not fit a simple model of neutral evolution. *D* and *Fs* were not significantly different from 0 in the other five SAMOVA groups, which suggests a neutral evolution. Consistently, mismatch distributions performed on all 29 populations and each of the 8 groups showed that the 29 populations pooled together and the groups Mt-C, Mt-G and Mt-F were compatible with the sudden expansion model (Fig. 4) with P_SSD_ = 0.500, 0.0794, 0.0841 and 0.103, respectively. The sudden expansion model was rejected for all other five populations groups.

We tested the hypothesis of a recent bottleneck or a recent population expansion for each of the 21 genotyped *B. dorsalis* populations using microsatellite data with either the SMM or the TPM models. Under the SMM model, a bottleneck event (heterozygote excess) was found for TC (P = 0.023) and GJ (P = 0.012). On the contrary, a significant heterozygote deficit (i.e., a sign of expansion) was detected in YY (P = 0.039), YK (P = 0.023), HA (P = 0.027), HC (P = 0.034) and JA (P = 0.019), suggesting a recent population expansion for these populations. However, under the more relaxed hypothesis of a certain proportion of mutations encompassing more than a single repeat unit (TPM), the results suggested that no populations had faced a bottleneck. The population expansion signal was confirmed in JA (P = 0.039) and HC (P = 0.043).

We also tested the hypothesis of a recent bottleneck or expansion for each of the 7 microsatellite SAMOVA groups. Under the SMM model, population groups Msat-A (P = 0.016) and Msat-E (P = 0.016) showed signs of recent significant bottlenecks, while groups Msat-C (P = 0.0195), Msat-F (P = 0.039) and Msat-G (P = 0.008) experienced a recent population expansion. When using the TPM model, no bottleneck was found for any of the 7 groups, but groups Msat-C (P = 0.039), Msat-E (P = 0.039), Msat-F (P = 0.039) still showed signs of recent expansion.

### Migration rate estimate

#### Mitochondrial data

Table S2 shows the amount of mutation-scaled immigration rate in both directions estimated using MIGRATE. Levels of migration rate ranged from 20.4 (from MM to HA) to 695 (from VP to VN). Asymmetric migration values were found from VP, GZ, CS, VN and TB to other populations. The two populations of Myanmar showed asymmetric migration to populations of western China (YR and YL). A similar situation was found from FX to populations of central China (HA, HC and JA). On the contrary, most Yunnan populations as well as GJ, LL, LM, NK, HC, HA and JA showed low migration rates towards other populations.

#### Microsatellite data

GENECLASS 2.0 was used to estimate the proportion of immigrants (m) into each population (see Table 7). The diagonal values of the assignment matrix indicate the average probability with which individuals were assigned to their own reference populations. The self-assignments probability values ranged from 0.50 (HC) to 0.67 (YK). Concerning the estimation of migration rates, the values ranged from 0 (from FX to HC) to 0.46 (FX to JA, GZ to NK). Interestingly, among 20 migration rates from VP and from GZ to other populations, 18 were over 0.10, whereas values of migration estimates towards VP and towards GZ were low. Similarly, relatively high estimates of migration rates were found from FX, GN, VN TB, TC and CS to other sites. On the contrary, very low migration rates were estimated from Myanmar (MM), from LL, from most locations in Yunnan (YY, YK, YD, YQ, YH, YR), from GJ and from HC as at least 15 estimates were below 0.03. Migration rates to Myanmar (MM) were also very limited whatever the potential population of origin. The same is true for migration estimates to HC.

**Table 7:** Mean assignment rates of individuals into (rows) and from (column) each population estimated by GENECLASS 2.0.

Population	MM	VP	VN	LL	CS	TC	TB	YY	YK	YD	YQ	YR	YH	GJ	GN	GZ	FX	NK	JA	HC	HA
MM	**0.59**	0.04	0.00	0.00	0.00	***0.06***	0.04	0.00	0.00	0.00	0.00	***0.07***	0.00	0.00	0.00	0.03	0.00	0.03	0.00	0.01	0.01
VP	0.00	**0.61**	***0.08***	0.00	0.03	0.02	0.03	0.01	0.01	0.00	0.00	0.00	0.00	0.00	*0.12*	*0.12*	*0.13*	0.04	0.03	0.00	***0.06***
VN	0.01	*0.28*	**0.60**	0.03	***0.08***	***0.08***	***0.08***	0.03	0.04	0.02	0.02	0.02	0.00	0.04	*0.11*	*0.11*	*0.11*	*0.1*	***0.09***	0.00	0.04
LL	0.01	*0.27*	*0.2*	**0.61**	*0.2*	*0.13*	*0.17*	0.01	0.04	0.00	0.03	0.03	***0.06***	*0.1*	***0.09***	***0.09***	*0.2*	*0.1*	*0.14*	0.01	0.03
CS	0.01	*0.2*	***0.07***	***0.05***	**0.59**	*0.12*	*0.17*	0.02	0.03	0.02	0.02	0.03	0.00	***0.07***	*0.14*	*0.16*	*0.14*	0.04	***0.08***	0.01	0.04
TC	0.03	*0.14*	***0.07***	0.02	*0.14*	**0.58**	*0.19*	0.02	0.02	0.01	0.01	0.01	0.02	0.02	***0.05***	*0.24*	***0.07***	0.08	0.03	0.01	0.03
TB	0.05	*0.13*	***0.07***	***0.05***	*0.15*	*0.16*	**0.62**	0.03	0.02	0.01	0.01	0.04	0.03	0.03	***0.06***	*0.22*	***0.05***	0.03	0.03	0.01	***0.06***
YY	0.02	*0.38*	*0.18*	0.02	*0.2*	*0.13*	***0.07***	**0.65**	***0.09***	***0.07***	***0.06***	0.01	0.03	0.01	*0.13*	*0.1*	*0.2*	0.02	*0.15*	0.00	0.03
YK	0.00	*0.13*	*0.1*	0.01	***0.08***	0.04	0.03	0.00	**0.67**	0.00	*0.23*	0.03	0.00	0.01	0.03	*0.15*	0.02	0.00	0.02	0.00	0.01
YD	0.02	*0.34*	*0.26*	0.02	*0.14*	*0.14*	***0.09***	*0.2*	0.03	**0.62**	0.01	0.01	0.01	0.01	0.02	*0.1*	*0.16*	0.03	*0.1*	0.00	0.02
YQ	0.00	*0.16*	*0.12*	0.02	***0.06***	***0.05***	0.03	0.01	*0.38*	0.00	**0.61**	0.04	0.00	0.03	***0.08***	*0.15*	***0.08***	0.05	***0.06***	0.00	0.04
YR	*0.32*	*0.19*	***0.08***	0.02	***0.08***	*0.2*	***0.09***	0.04	0.03	0.01	0.04	**0.57**	0.01	0.01	0.01	*0.16*	*0.11*	0.08	***0.09***	0.01	***0.09***
YH	0.02	*0.34*	*0.17*	*0.1*	*0.26*	*0.11*	*0.23*	***0.06***	0.03	0.04	0.02	0.04	**0.66**	0.06	*0.22*	*0.27*	*0.19*	0.03	*0.11*	0.00	***0.07***
GJ	0.00	*0.14*	*0.15*	0.04	*0.14*	***0.05***	*0.1*	0.04	0.04	0.00	***0.05***	0.00	0.00	**0.59**	*0.13*	*0.17*	*0.14*	0.07	*0.1*	0.01	***0.06***
GN	0.01	*0.55*	*0.22*	***0.07***	*0.18*	***0.06***	*0.15*	***0.06***	***0.05***	0.00	0.04	***0.05***	0.01	0.06	**0.55**	*0.18*	*0.36*	*0.27*	*0.18*	0.01	***0.08***
GZ	0.02	*0.24*	***0.07***	0.00	0.01	***0.09***	***0.08***	0.00	0.01	0.00	0.00	0.04	0.00	0.00	***0.08***	**0.58**	0.00	0.03	*0.13*	0.04	*0.13*
FX	0.01	*0.44*	*0.15*	0.04	*0.17*	***0.09***	***0.07***	0.02	0.03	0.00	0.03	0.02	0.00	0.05	*0.19*	*0.11*	**0.57**	*0.14*	*0.46*	0.01	***0.06***
NK	0.02	*0.32*	***0.09***	0.00	0.02	0.1	*0.11*	0.00	0.01	0.00	0.01	0.03	0.00	0.03	*0.12*	*0.46*	*0.15*	**0.55**	*0.16*	0.01	***0.07***
JA	0.01	*0.3*	***0.08***	0.01	***0.07***	0.02	0.02	0.03	0.02	0.00	0.02	0.00	0.00	0.02	*0.10*	*0.16*	*0.46*	*0.16*	**0.53**	0.01	*0.14*
HC	0.00	0.01	0.00	0.00	0.00	0.03	0.02	0.00	0.00	0.00	0.00	0.01	0.00	0.00	0.00	*0.12*	0.00	0.01	0.00	**0.50**	*0.31*
HA	0.01	*0.3*	***0.08***	0.01	***0.07***	0.02	0.02	0.03	0.02	0.00	0.02	0.00	0.00	0.02	*0.11*	*0.16*	*0.40*	0.16	*0.23*	0.01	**0.54**

Values in bold indicated the proportions of individuals assigned to the same population. Values of m above 0.05 are bold italics and above 0.1 are italics.

## Discussion

In this study, we obtained data using both mitochondrial and nuclear DNA markers of an extensive sampling of *B. dorsalis* in Asia to unravel the patterns of genetic differentiation of an important insect pest over a large area of its geographical distribution, including its potential invasive range. Our results go well beyond previous studies [7], [17], [18], [20], because of the concomitant use of both maternally and bi-parentally inherited markers, and because the sampling procedure allowed for a clearer picture to be drawn of the genetic structure and possible migration pathways of the fly in Asia by combining regions that were so far analyzed separately.

### Main patterns of genetic structure in China and Southeast Asian countries

#### A significant geographical pattern revealed by all markers

Mitochondrial and microsatellite data both revealed a weak but significant genetic structure that corresponds to a geographic pattern (see Table 4 and Fig. 2, 3). The limits of most population groups were actually similar with both markers (1- Myanmar and Western Yunnan; 2- Laos and Southern Yunnan; 3- Thailand and Cambodia; 4- Vietnam and Southern China; 5- Central China (HC & HA)). In some cases, the exact limits of groups were different when assigned from mitochondrial sequences or from microsatellite loci. For instance, the two easternmost Chinese populations (JA and FX) were in a separate cluster for mtDNA but were included in an existing group (Vietnam and Southern China) based on microsatellite data. The main differences between markers concerned the genetic structure within Yunnan, where mtDNA clearly differentiated the southern from the northern populations, while microsatellites revealed an East-West differentiation. Such limited discrepancies could be due to the different evolutionary patterns of both markers as mtDNA corresponds to the maternal lineage, evolves quite slowly as compared to microsatellites, and is more prone to genetic drift.

Our data allowed for the identification of some patterns that did not appear previously, probably because of the limited sampling studied in previous papers. Some of the identified groups (in particular Myanmar, Laos and central China) correspond either to geographically distant sites, sometimes at the limit of the fly's range (similar to the sample from Bangladesh in [7]), or to natural topography. The role of topography in shaping the genetic structure of *B. dorsalis* was already discussed in previous studies [7], [18], [20], and is now confirmed for both types of markers over a fairly large sampling range. For instance, the (Myanmar and Western Yunnan) group is isolated from the rest of the distribution range by the Hengduan mountains and by rivers (namely Nujiang, Lancangjiang and Yuanjiang), which probably strongly reduce gene flow. Similarly, the (Laos and Southern Yunnan) group is located on high plateaus that are relatively isolated from the other sampling sites, the corresponding populations being connected through natural south-north oriented corridors created by mountains running from Yunnan to Laos. Further, in Yunnan, microsatellite analyses identified two pairs of populations (YY & YD; YK & YQ) that actually fall in two natural gorges created by mountains and rivers forming natural routes for population dispersal. Moreover, monsoon current originating from the Bengal fjord [60]–[62] and blowing from southwest to northeast also facilitate the dispersal of flies from south to north [18]. Concerning the relative differentiation of central China (HA and HC, located in Hunan and Hubei provinces respectively), their isolation could be explained by topography (transition zone of plateau and plain, surrounded by high mountains, namely Xuefeng, Nanling and Luoxiao mountains [63]). Moreover, one of the sites (HC) corresponds to the northern limit of the fly's distribution in China (Latitude 32°2′N) [64], which most probably also highly limits natural dispersal.

On the other hand, extensive gene flow was found between populations of Vietnam and southern China, both with mtDNA and microsatellite markers. This lack of differentiation can be associated with the smooth topography and continuous plant cultivation. Mango, the major host-plant of this fruit fly, is planted widely in these regions [63], [65]. Continuous host resources together with the absence of natural barriers to gene flow promote genetic homogenization. Moreover, the monsoon currents blow from Pacific Ocean [63] and facilitate the dispersal of the fly between these regions.

#### A weak signal blurred by a complex distribution of diversity

In spite of the clear geographical patterns identified by both markers, one should keep in mind that the underlying structure is actually weak, as it explains only 10–12% of the molecular variance. Up to 82% of this variance is explained by within- population genetic diversity in both markers (Table 4). The network of mitochondrial haplotypes reflected a random pattern and did not allow identification of phylogenetic haplogroups, as the haplotypes shared between geographically close populations were unrelated. Interestingly, Wan et al. [20], using three mitochondrial genes, also found a weak genetic structure in China and complex, star-like haplotype networks. In the same manner, using microsatellite markers, some populations groups showed high levels of admixture between the clusters identified with STRUCTURE. A very similar picture was obtained by Aketarawong and colleagues in South-East Asia [7], yet from different populations and different microsatellite loci. Moreover, no IbD pattern was observed in any of the two markers. We may interpret these results as evidence of repeated long distance migration events within geographical regions. This pattern is actually common in mobile insects, such as the South American fruit fly *Anastrepha fraterculus* [66], the fruit flies *Bactrocera invadens* [23] and *Bactrocera cucurbitae* [67] or the migratory dragonfly *Anax junius* [68]. As *B. dorsalis* is a phytophagous fruit fly associated with many cultivated plants, this complex and random genetic structure could also be due to human-aided dispersal (plant exchanges) and range expansion (see below). Even though we did not find any significant genetic structure due to host groups (Table 6), we suggest that the high degree of polyphagy of this insect species has favored its regional dispersal. Commercial exchanges of various host plants carrying eggs or larvae may also be involved. By contrast, *Bactrocera oleae*, a monophagous fly which is tightly linked to cultivated olive trees and wild relatives [11], showed strong phylogeographic and IbD genetic patterns. The repeated long distance migration suggested for this fly means that human mediated activities influence the genetic pattern of this fly. In summary, the combination of geographic patterns and barriers, and of human mediated effects (fruit transportation and trade, people immigration, tourism) probably acted together to shape the genetic structure of *B. dorsalis* in Asia.

### What about migration pathways and potential source areas for *B. dorsalis*?

In most cases of introduction of alien pest species outside of their native range, invasive and expanding populations can be identified from genetic data by analyses of diversity indices. Assignment tests can also help identifying the source populations if the invasion is relatively recent and if the native populations are sufficiently structured. Introduced populations are expected to show signs of founder events because they usually originate from a reduced number of individuals; such a situation results in reduced diversity and heterozygosity indices [11]. This is actually the case for *B. dorsalis* in Hawaii [7]. In other cases, the invasion can result in gradients of diversity, such as when a population from a nearby source area actually disperses into the unoccupied area in a gradual, stepping-stone process. In such cases, the genetic variation present in the source population will gradually be lost as the distance to newly established sites increases [69]. The number of private alleles in the newly founded population is moreover necessarily low, because all alleles in the invaded regions were brought from the natural range, except when mutation occurs. Such a pattern was actually found for *B. oleae*, which is expanding around the Mediterranean and in the New World from an African origin [11]. The pattern also matches the results found for *B. cucurbitae* invading East Asia from its central Asia source area [24].

Concerning *B. dorsalis*, previous studies already pointed out a high level of genetic diversity in most studied regions, namely South-East Asia [7], China [20] or Yunnan province [17], [18], making the identification of native and invasive ranges difficult. There have been several hypotheses about potential source areas of *B. dorsalis*, based on ecological and molecular data. Taiwan was first considered as a plausible source [8], but this possibility was rejected by Aketarawong et al. [7]. Clark et al. [6] hinted that the fly could be native to Southeast Asia based on investigation of the *B. dorsalis* species complex. Finally, regions along the southeast coast of China were hypothesized to represent the source area of *B. dorsalis* [7], [20]. In the present study, we combined both mitochondrial and nuclear DNA markers and a large area of sampling from other Southeast Asian countries to central China to infer the colonization pathways of *B. dorsalis*.

All *B. dorsalis* populations and regions showed both private microsatellite alleles and mitochondrial haplotypes, and diversity indices were never drastically reduced. As mentioned above, the patterns of genetic structure were complex, and multiple long-distance migration events have blurred the picture. It is today very difficult to disentangle the evolutionary and colonization history for this species. Nonetheless, all markers showed that the maximal diversities were found in Myanmar and Vietnam. Diversities indices were also high in Thailand and Cambodia. Consistent with the studies by Wan et al. and Aketarawong et al. [7], [20], the most diverse population of China was located in Guangdong. On the contrary, the populations from Yunnan only had sub-samples of mitochondrial haplotypes, and the minimal values of allelic richness. In the same manner, central and southern populations of China (GN, GJ, JA, HC, HA, NK) also showed lower indices of genetic diversity (haplotype numbers, unique haplotypes and private alleles). Moreover, migration estimates were consistent across markers and suggested that gene flow preferentially occurred from Vietnam, Cambodia and Guangdong (GZ) to other regions, while it was very low from Yunnan to elsewhere. We might consider these results as evidence that the tropical regions of Southeast Asia and the southern coast of China fall within the native range of *B. dorsalis*, and that they expanded northward up to central China and eastward to Yunnan. This is consistent with a previous study based on ecological data [8]. Based on these results, we suggest that Yunnan is a contact zone that has been colonized from different sources and excluded the possibility that Yunnan could al within the native range. This explains why previous studies [17], [18] had found significant geographic structure and a globally high genetic diversity in Yunnan. The westernmost part of Yunnan is genetically similar to the nearby populations of Myanmar, while the southern regions of Yunnan are linked to Laos along the natural corridors created by mountains and rivers. Thailand and Cambodia also contributed to the source for Yunnan by human transportation. Regions along the southern coast of Vietnam and China probably served to colonize mainly the central region of China (GN, GJ). South coastal region of China perhaps also colonized central part of China (HA, HC) and part of central Yunnan.

Results of demographic analyses obtained using both molecular markers also supported this scenario. Populations of Southeast Asia and southern China did not experience any recent population expansion or contraction as shown by neutrality tests and bottleneck analysis. This is consistent with the hypothesis that these regions correspond to the stable, native area of *B. dorsalis*. On the contrary, mitochondrial and microsatellite data suggest that populations located at the central and northern range of the species distribution in China experienced recent population expansions, which is consistent with the results obtained by Wan et al. [20] in Central China. *B. dorsalis* populations probably can easily move northward and gradually expand into these new territories. Some populations in western China also experienced recent expansions. Our results suggest that demographic expansion of *B. dorsalis* is still an on-going process.

Certain biological characteristics of *B. dorsalis*, such as its wide host range, climatic tolerance and high dispersal capacities [35] have probably facilitated its range expansion over the years. The risk exists that other regions will soon become suitable for *B. dorsalis* establishment due to global warming. Ecological data suggest that the potential geographic distribution of this species has increased to 35°N [70]. More attention should be paid to this pest species, and improved quarantine and sanitary control measures need to be implemented to avoid or slow the rate of new invasions.

## Supporting Information

Figure S1 **Haplotype network of the 73 haplotypes found.** The size of each ellipse is proportional to the number of individuals having a particular haplotype, which is given within the ellipse. The empty circles correspond to missing intermediate haplotypes. The proportion of individuals belonging to each of the eight SAMOVA groups is represented by a color code (see text for details).
(TIF)

Figure S2 **Values of F_CT_ and LnP(D) for SAMOVA groups and STRUCTURE clusters.** A: F_CT_ values from K = 2 to 10 based on mitochondrial SAMOVA results. B: F_CT_ values from K = 2 to 10 based on microsatellite SAMOVA results. C: Log-likelihood probability LnP(D) of the number of inferred clusters (K) as a function of K using STRUCTURE, for K = 2 to 13, with 10 independent runs for each K.
(TIFF)

Table S1 **Pairwise F_ST_ values based on mitochondrial sequences (below diagonal) and microsatellite loci (above diagonal).** Some of the values were not significantly different from zero (ns)
(XLS)

Table S2 **Effective immigration rate between populations pairs estimated from mitochondrial data using MIGRATE.** Instances of asymmetrical gene flow are indicated in bold. The source population is indicated in columns, the target population in row.
(XLS)
