# Supplementary figures and images for: Genetic Structure and Inferences on Potential Source Areas for Bactrocera dorsalis (Hendel) Based on Mitochondrial and Microsatellite Markers
Source: PLoS One. 2012 May 16;7(5):e37083. doi: 10.1371/journal.pone.0037083 (PMC3353900; doi:10.1371/journal.pone.0037083)

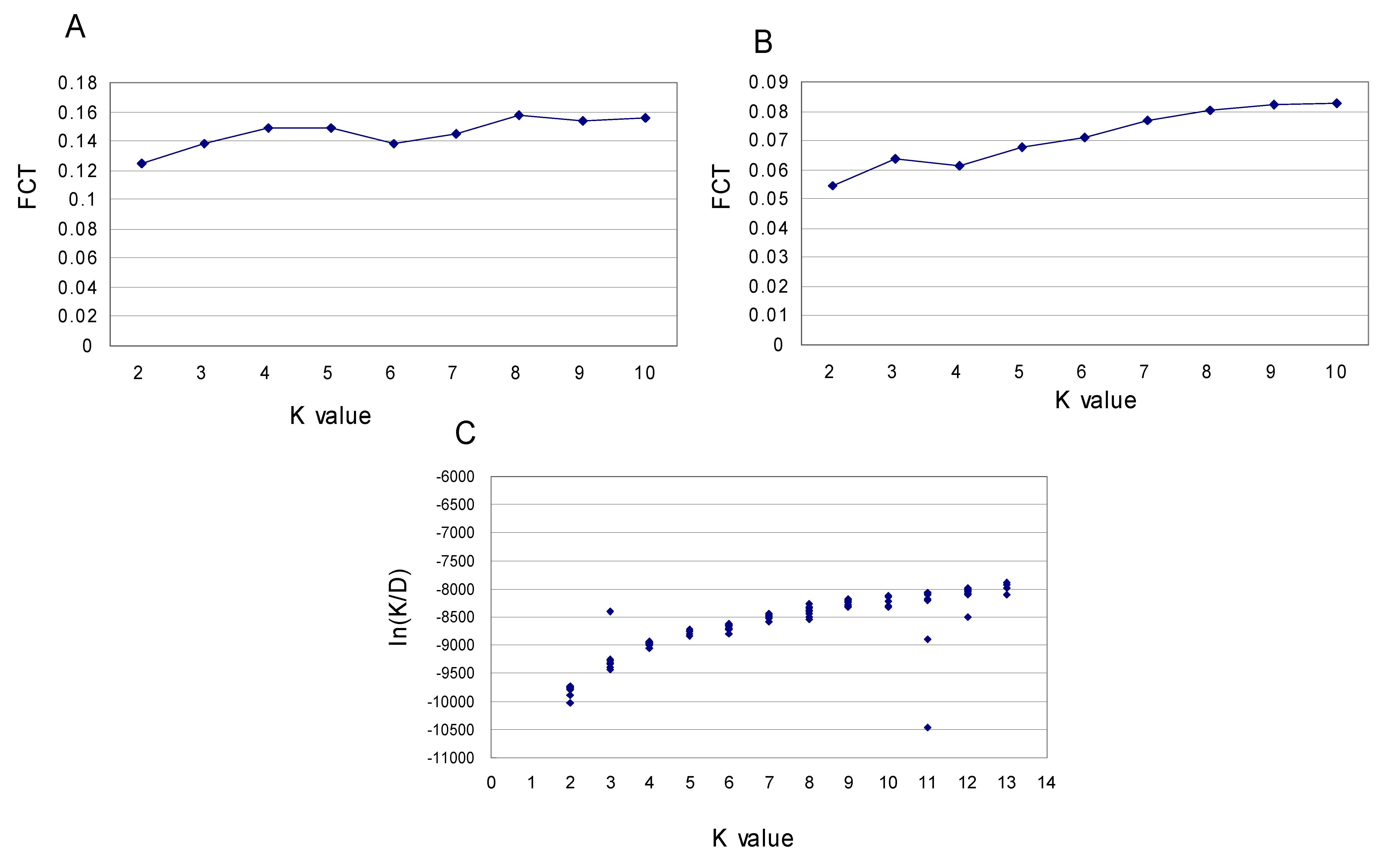

Supplement: Figure S2 — Values of FCT and LnP(D) for SAMOVA groups and STRUCTURE clusters. A: FCT values from K = 2 to 10 based on mitochondrial SAMOVA results. B: FCT values from K = 2 to 10 based on microsatellite SAMOVA results. C: Log-likelihood probability LnP(D) of the number of inferred clusters (K) as a function of K using STRUCTURE, for K = 2 to 13, with 10 independent runs for each K. (TIFF) [file pone.0037083.s002.tiff]
